# Cytokine Attenuation and Free Radical Scavenging Activity of a New Flavanone7,4′-Dihydroxy-3″,3″-Dimethyl -(5,6-Pyrano-2″-One)- 8- (3‴,3‴-Dimethyl Allyl)- Isolated from *Mallotus philippensis*: Possible Mechanism for Its Anti-Inflammatory Activity

**DOI:** 10.1371/journal.pone.0167294

**Published:** 2016-12-12

**Authors:** Waseeem Rizvi, Mohd Fayazuddin, Ompal Singh, Syed Shariq Naeem, Shagufta Moin, Kafil Akhtar, Anil Kumar

**Affiliations:** 1 Department of Pharmacology, Jawaharlal NehruMedical College, Aligarh Muslim University, Aligarh, India; 2 Chemical Research Unit Department of research in Unani Medicine Aligarh Muslim University, Aligarh, India; 3 Department of Biochemistry, Jawaharlal NehruMedical College, Aligarh Muslim University, Aligarh, India; 4 Department of Pathology, Jawaharlal NehruMedical College, Aligarh Muslim University, Aligarh, India; Indian Institute of Integrative Medicine CSIR, INDIA

## Abstract

*Mallotus philippensis* L.(MP) commonly known as Kamala tree in Hindi,is a small to medium-sized monoecious tree.The objective of the study was to evaluate the anti-inflammatory activity of MPand a new flavanoneisolated from it by using in vivo models of inflammation.Albino wistar rats of either sex weighing 150-200g were used. Seven groups were made (n = 6), namely normal control group (normal saline, 1 ml/kg), standard control group (acetylsalicylic acid, 100 mg/kg), methanol crude extract (300 and 500 mg/kg), ethylacetate fraction (300 and 500 mg/kg) and active compound 4 (new flavanone, 50 mg/kg). The anti-inflammatory activity was studied using carrageenan induced paw edema method and cotton pellet granuloma method. Levels of cytokines (TNF-α, IL-1and IL-6) and activity of antioxidant enzymeslike catalase and glutathione peroxidase were estimated. It was found that the methanol extract, ethylacetate fraction and Flavanonedemonstrated significant reduction in paw edema in carrageenan induced paw edema method as compared to control. They also diminished the serum TNF-α, IL-6 and IL-1 levels. Significantly attenuated the malondialdehyde levels and increased the activities of catalase and glutathione peroxidase in paw tissue. Similarly there was asignificant decrease in granuloma formation in cotton pellet induced granuloma method. In conclusion, MP extracts and the newflavanonepossess anti-inflammatory activity and this might be due to the inhibition of various cytokines and increased free radical scavenging activity.

## Introduction

*Mallotus philippensis* L.(MP) is a large woodymultipurpose medicinal tree belongs to the family Euphorbiaceae.MP’s natural habitat is in the foothills of the Himalayas in Northern India, Nepal, Southern China and South East Asia including Thailand, Myanmar, and Malaysia.[1] For many centuries root, bark, leaves and glands/hairs (red powder covering the fruits) of MP is used in Ayurveda for the treatment of a variety of disease conditions. In folklore, decoctions of the bark of MP has been used to treat typhoid and meningitis. [2, 3] The glands and the hair of the fruits are used as a purgative and in the treatment of worm infestation. In addition, the leaves are used topically to treat different types of skin and superficial wound infections. [4]MP is reported to possess anthelmintic activity against tapeworm infestation in rats. [5]Antiallergic activity has also been reported in two phloroglucinol derivatives isolated from the fruits of MP. Its hepatoprotective activity was demonstrated on the primary cultured hepatocytes treated with carbon tetrachloride [6] while antioxidant activity was demonstrated using *in vitro* tests like DPPH and total phenolic content ofvarious parts of the plant.[7]The methanol extract of MP is shown to have bactericidal activity against *S*.*aureus*, *S*.*saprophyticus*, *Bacillus*,*E*.*coli* and antifungal activity against *Microsporum gypseum*.[8] Ishii *et al* have demonstrated anti-inflammatory activity of *Mallotus japonicas* by inhibition of proinflammatory cytokines. [9] The antiinflammatory activity of drugs in allopathic medicine is due to the virtue of cycloxygenase (COX) which serves as the enzyme for the synthesis of inflammatory mediatorslike prostaglandins. [10,11,12] Apart from prostaglandins, leucotrienes, interleukins (IL) and tumor necrosis factor (TNF) also play an important role in the development of inflammatory and immune response.[13,14,15]. Although MP has been used traditionally in various inflammatory conditions but the scientific data validating its medicinal properties is scarce. Hence, the present study was designed to evaluate the antiinflammatory property of MP and its isolated compounds.

## Material and Methods

### Plant Material and Extraction

The plant MP was purchased from local market in Aligarh, India in June 2012. It was identified by Dr. Athar Ali Khan, Taxonomist, Department of Botany, Aligarh Muslim University (A.M.U). Aligarh and Dr. RifatAfridi, Research Officer (pharmacognosy) Regional Research Institute Unani Medicine, Aligarh. A voucher specimen bearing the number 433 was deposited in the herbarium of Department of Botany, A.M.U., Aligarh, India.

The fruits of the MP were shade dried and powdered by an electric grinder. Exhaustively extracted using Soxhletextractor with petroleum ether, benzene, chloroform, ethyl acetate and methanol (inthat order). The solvent was removed by distillation. The petroleum ether and benzene extracts were concentrated under reduced pressure to yield a greenish gummy mass. To ascertain the number of compounds a thin layer chromatography (TLC) examination was done. The petroleum ether and benzene extracts showed similar behaviour on TLC, hence they were mixed together. The mixed petroleum ether- benzene and ethylacetate fraction was further subjected to silica gel column chromatography. The column was eluted successively with petroleum ether, petroleum ether-benzene (differential relative concentrations), benzene, benzene-ethyl acetate(differential relative concentrations), ethyl acetate, ethyl acetate-methanol(differential relative concentrations) and finally with blank methanol (S1 File). These fractions were purified by repeated column chromatography followed by crystallization to yield 4 active compounds (A1 to A4). Ethyl acetate-methanol (1:1) fraction yielded a previously unknown flavanone ***7*,*4***^***′***^***-Dihydroxy-3***^***′′***^**,*3***^***′′***^***-dimethyl -(5*,*6-pyrano-2***^***′′***^***-one)- 8- (3***^***′′′***^**,*3***^***′′′***^***-dimethyl allyl)-flavanone* (Compound- A4).** A crude extract of methanol was also prepared for further evaluation.The yield of the petroleum ether, benzene, chloroform, ethyl acetate and methanol was 16.3%, 8.2%, 7.8%, 2.1% and 2.2%, respectively. The yield of crude methanol extract was 7.4%.

### Drugs and Chemicals

Acetylsalicylic acid (Purity 97.8%, Reckitt Benckiser, India), propylene glycol (BDH, Mumbai) and carageenan(Sigma Chemicals, USA) rat TNFα ELISA kit (Biomolecular Integrations), rat IL-6 ELISA kit (Koma Biotech, Korea), rat IL-1α ELISA kit (Boster Biological Technology, LTD) were used in the study.

### Animals

Adult albino Wistar rats of either sex weighing 150-200g were procured from the Central Animal House, Jawaharlal Nehru Medical College (JNMC), A.M.U. They were housed in polypropylene cages at ambient temperature (25± 2°C), relative humidity (55 ± 5%) and 12-hr light-dark cycle. Animals had free access to standard pellet diet and water *ad libitum*. The study protocol was approved by the Institutional Animal Ethics Committee, J.N.M.C., A.M.U, Aligarh (India) (401/CPCSEA). All the experiments were carried out according to the animal handling guidelines of Committee for the Purpose of Control and Supervision of Experiments on Animals (CPCSEA), Ministry of Environment, Forest and Climate change, Government of India.

### Experimental design

Animals were distributed into seven groups of six animals each (n = 6). Group-I served as control and was given normal saline 1 ml/kg, Group-II served as standard and was given acetylsalicylic acid(100mg/kg), GroupsIII and IV were given methanol extract of MP (ME) in a dose of 300 mg/kg and 500mg/kg, respectively.Groups V and VI were given ethylacetate fraction (EF) in a dose of 300 mg/kg and 500mg/kg, respectively. Group VIIwas given isolated compound(A4) in a dose of 50mg/kg.

### Carrageenan induced paw edema method

It is one of the most commonly employed methodsfor the screening of the drugs for acute inflammation [16]. In all the groups, acute inflammation was induced in the subplantar region ofthe right hind paw with 0.1 ml of freshly prepared 1% suspension of carrageenan in normal saline subcutaneously after 1 hour of administration of test compounds.The paw was marked at the level of the lateral malleolus and was immersed every time up to this mark. The paw volumes were measured at 0h, 1h, 2h, and 3h after the carrageenan injection using digital plethysmometer (Orchid scientific, India).

The percentage inhibition of paw edema at each time interval was calculated by using the following formula:-

$$Percentage\;inhibition=\frac{(Vt-Vo)control-(Vt-Vo)\;treated}{(Vt-Vo)\;control}\times 100$$

Where,

Vo = Paw volume of test/control group at 0 hr

Vt = Paw volume of test/control group at that particular time interval.

At the end of the experiment,the ratswere sacrificed by overdose of pentobarbitone and the plantar tissue from the right hind paw was removed and homogenised with phosphate buffer solution four times of their respective volume. Then the homogenate was centrifuged and the supernatant was obtained and stored at −40°C for the antioxidant enzyme activity assays.

### Cotton pellet induced granuloma method

This method is used for the screening of drugs active against subacute inflammation [17]. In brief, under anaesthesia with aseptic precautions two sterilized cotton pellets (10mg) were implanted subcutaneously on either side of the lumbar region in each rat. The incisions were sutured by silk 2.0 sutures and betadine solution was applied to prevent any infection. The respective test compounds were given every day for 7 days (including the day of implantation of the cotton pellets) in all the groups. On the 8^th^ day, animals were anaesthetized by 50mg pentobarbitone sodium I.P. with 0.05mg/kg I.P. of atropine (to decrease secretions) and the cotton pelletswerecarefully excised out. The pelletsweredried at 60°C for 24 h. The dry weight of the granuloma was calculated as the difference in the dry weight of the cotton pellets recorded before and after implantation. The incisions were again sutured by silk 2.0.The animals were returned to the Central Animal House for rehabilitation and monitored (6 hourly on the first day, then every day till healing).

Percentage inhibition was calculated by using the following formula.

$$Percent\;inhibition=\frac{WC-WT}{WC}\times 100$$

Where,

WC = Dry weight of the cotton pellets in control animal.

WT = Dry weight of the cotton pellets in drug treated animals.

### Estimation of Cytokines TNF-α, IL-6 and IL-1 in serum

After completion of the carrageenan-induced paw edema experiment, the rats were anesthetized and blood samples were collected from orbital sinus. The serum was separated by centrifugation and was stored at −40°C until further use.TNF-α, IL-6 andIL-1 from each sample were measured in duplicate with highly sensitive rat TNF-α Elisa Kit (Biomolecular Integrations), rat IL-6 Elisa Kit (Koma Biotech), rat IL-1α Elisa Kit(Boster Biological Technology LTD) respectively, according to the manufacturer’s instructions. The results were expressed as pg/ml of serum.

### Determination of Tissue Lipid Peroxidation

Malondialdehyde (MDA) was estimated in the foot by thiobarbituric acid reacting substance(TBARS) method.[18] Briefly,at high temperature MDA reactswith thiobarbituric acid at acidic pH resulting in the formation of a red complexTBARS. The absorbance of TBARS was determined at 532 nm using a double beam UV- VIS spectrophotometer.The results were presented as nmol/mg of protein.

### Determination of Antioxidant Enzyme Activity

Two enzymes, Glutathione peroxidise (GPx) and Catalase (CAT) were assessed for *in vivo* antioxidant activity in the serum. The GPx in the presence of H_2_O_2_ reduces it to water while catalyzing the reaction of GSH (Glutathione Reduced) turning into oxidized glutathione (GSSG). The GSSG formed is reduced again to GSH by the glutathione reductase reaction using NADPH as the reducing substrate. There is a decrease in absorbance during the oxidation of NADPHto NADP+ and this is measured by a spectrophotometer at 340 nm for the calculation of the GPx activity. [19] The results were given as U/mg protein.

CAT activity estimation was done by the reduction of 10mM H_2_O_2_ 20mM of phosphate buffer (pH 7) by free radical present in serum and was monitored by measuring the absorbance at 240 nm. [20] The activity was calculated by using a molar absorption coefficient, and the enzyme activity was defined as nanomoles of dissipating hydrogen peroxide per mg of protein per minute. Protein concentration was measured by Lowry method [21] using the bovine serum as a standard. The enzyme activities were expressed as units of enzyme activity per mg of protein.

### Histological Examination

Under deep anaesthesia biopsies of the paws were taken 3hours after the injection of carrageenan. The tissue slices were fixed in 10% neutral-buffered formaldehyde, dehydrated in graded ethanol and were embedded in paraffin. 5 μm thick slices were sectioned and were stained with hematoxylin and eosin. All samples were observed and photographed by Olympus microscopy. Tissue slices were randomly chosen from carrageenan, Acetylsalicylic acid,ME (500 mg/kg),EF treated (500 mg/kg) and A4(50mg/kg) groups. The numbers of neutrophils were counted in each scope using 400x magnifications(average count from 5 scopes of every tissue slice).

### Statistical analysis

All the values are expressed as Mean ± SEM. Statistical significance was calculated by one way ANOVA followed by post hoc Dunnett’s multiple comparison test. p<0.05 was considered to be statistically significant.

## Results

### Carrageenan induced paw edema

Acetylsalicylic acid (100 mg/kg) significantly decreased the paw edema at 1h (p<0.001) and 3h (p<0.001) as compared to control (Group I). The percentage inhibition of paw edema after giving acetylsalicylic acid was 59.58% and 87.06% at 1h and 3h respectively. Both ME and EF treated groups demonstrated a significant decrease in paw edema compared to control group (p <0.001). MEshowed a significant reduction in paw edema both at 1h and 3h whencompared to control and the percentage inhibition of edema was 38.3% (p<0.05) and 69.42%(p<0.001) at 1h and 3h, respectively with 300mg/kg. Whereas at 500mg/kg dose it was 51.07% (p<0.001) and76.48% (p<0.001) at 1h and 3h, respectively. EFalso showed significant reduction in paw edema both at 1h and 3h compared to control and percentage inhibition of edema was found to be 25.54% and 51.77%(p<0.001) at 1h and 3h, respectively with 300mg/kg and was 31.91% (p<0.01) and 63.52%(p<0.001) at 1h and 3h, respectively forthe dose of 500mg/kg.(Table 1)

**Table 1:** Effect of *M*. *philippensis* on Carrageenan induced paw edema.

Groups	Paw volume at different time interval (in ml)				Inhibition of edema(%)	
	0 hour	1 hour	2hour	3hour	1hour %	3hour
**Normal Control (Normal saline 1ml/kg)**	0.80+.37	1.27±.046	1.40±.045	1.65±.058	-	-
Acetylsalicylic acid **(100 mg/kg)**	0.82±.035	1.01±.026**	1.04±.043**	0.93±.035**	59.58	87.06
**ME (300 mg/kg)**	0.75±.025	1.04±.043**	1.09±.045**	1.01±.031**	38.3	69.42
**ME (500 mg/kg)**	0.76±.035	0.99±.043**	1.03±.049**	0.96±.037**	51.07	76.48
**EF (300 mg/kg)**	0.82±.025	1.17±.035**	1.24±.046**	1.23+.083**	25.54	51.77
**EF (500 mg/kg)**	0.86±.021	1.01±.01**	1.25±.032**	1.17±.029**	31.91	63.52
**A 4 (50 mg/kg)**	0.72±.016	0.92±.024**	0.94±.032**	0.84±.040**	57.45	85.89

Values are expressed as Mean±S.E.M.

** indicates p <0.001 when compared to the control group

ME- Methanol extract of *M*. *philippensis*, EF-Ethylacetate fraction of *M*. *philippensis*, A 4– Active compound of *M*. *philippensis*.

The Group VII given novel flavanone(A4) showed a significant reduction in paw edema. At 1h the percent inhibition of the paw edema was found to be 57.45% (p<0.05) while it was raised to a highly significant value of 85.89% (p<0.001) at 3h, which was comparable to control group of acetylsalicylic acid (87.06%, p>0.05). (Table 1)

### Cotton pellet induced granuloma

In Cotton pellet induced granuloma method, acetylsalicylic acid significantly inhibited the granuloma formation by 70.68% (p<0.001) when compared to control. Whereas ME significantly inhibited the granuloma formation by 48.19% (p<0.001) and 60.83% (p<0.001) compared to control group, in the doses of 300 mg/kg and 500 mg/kg, respectively. EF significantly decreased granuloma formation by 29.95% (p<0.05) and 40.74% (p<0.001) in the doses of 300 mg/kg and 500 mg/kg, respectively.

**Fig 1:**
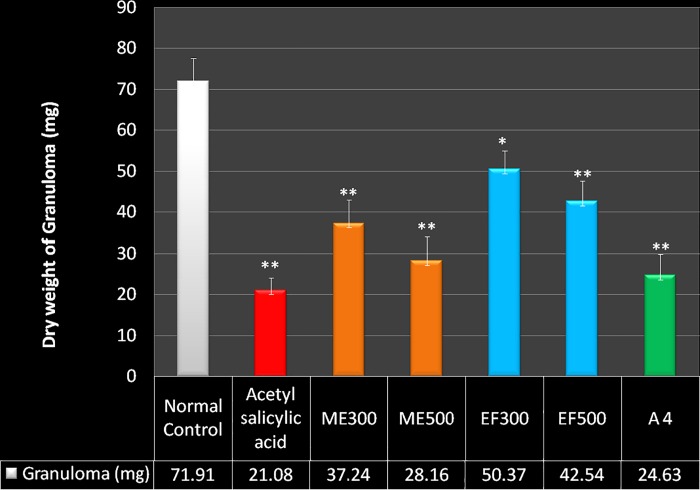
Effect of *M*. *philippensis* on dry weight of cotton pellet granuloma. Values are expressed as Mean±S.E.M. *indicates p<0.05, ** indicates p <0.001 when compared to the control group Aspirin-100mg/kg, Normal Control- Normal saline 1ml/kg, ME300—Methanol extract 300mg/kg, ME500-Methanol extract 500mg/kg, EF300-Ethylacetate fraction 300 mg/kg, EF500-Ethylacetate fraction 500 mg/kg, A 4– Active compound of *M*. *philippensis* (50mg/kg),.

A4 showed significant reduction of the granuloma formation by 65.21% (p<0.001) compared to control group and was comparable to acetylsalicylic acid treated group (Fig 1).

### Cytokines TNF-α, IL-6 and IL-1 in serum

As shown in Table 2, there was decrease in all the three serum cytokines (TNF, IL-6, IL-1) in ME treated groups at both 300mg/kg (p<0.05) and 500mg/kg(p<0.001) doses whereas in EF treated groups significant decrease was seen only at 500mg/kg(p<0.05) dose.A4 also showed a significant(p<0.001) reduction in all the three serum cytokines

**Table 2:** Effects of *M*. *philippensis* on serum cytokines.

GROUPS	TNF α(pg/mL)	IL- 6 (pg/mL)	IL- 1 (pg/mL)
**Normal Control (Normal saline 1ml/kg)**	707.50±65	605.25±43	252.50±26
Acetylsalicylic acid **(100 mg/kg)**	400.25±48**	282.00±33**	124.00±13**
**ME (300 mg/kg)**	522.50±36*	479.75±34*	179.00±14*
**ME (500 mg/kg)**	459.75±33**	406.52±29**	167.25±22*
**EF (300 mg/kg)**	600.00±45	552.52±31	221.75±17
**EF (500 mg/kg)**	560.00±19*	484.50±23*	178.75±30*
**A 4 (50 mg/kg)**	406.25±29**	353.25±27**	141.50±15**

Values are expressed as Mean±S.E.M.

*indicates p<0.05

** indicates p <0.001 when compared to the control group

ME- Methanol extract of *M*. *philippensis*, EF-Ethylacetate fraction of *M*. *philippensis*, A 4– Active compound of *M*. *philippensis*.

### Antioxidant enzymes and MDA assay

There was a significant decrease in MDA levels in both ME (p<0.001) and EF (p<0.001) treated groups and increase in both antioxidant enzyme activities (CAT and GPx) in all the groups treated with ME (p<0.001) and EF (p<0.001).

A4 showed a significant reduction in MDA levels (p<0.001) while an increase in both the antioxidant enzyme activities was observed (CAT and GPx) (p<0.001). (Table 3)

**Table 3:** Effects of *M*. *philippensis* on MDA, CAT and GPx activities.

GROUPS	MDA(nmol/mg protein)	Catalase (U/mg protein)	GPx(U/mg protein)
**Normal Control (Normal saline 1ml/kg)**	1.60±.08	3.22±.05	9.60±.27
Acetylsalicylic acid **(100 mg/kg)**	0.74±.03**	4.60±.11**	16.72±.18**
**ME (300 mg/kg)**	1.11±.06**	4.02±.05**	12.12±.12**
**ME (500 mg/kg)**	1.01±.05**	4.35±.04**	13.85±.17**
**EF (300 mg/kg)**	1.35±.04**	3.41±.05*	11.02±.02*
**EF (500 mg/kg)**	1.24±.03**	3.80±.05**	11.91±.13**
**A 4 (50 mg/kg)**	0.81±.06**	4.50±.19**	14.5±.37**

Values are expressed as Mean±S.E.M.

*indicates p<0.05

** indicates p <0.001 when compared to the control group

ME- Methanol extract of *M*. *philippensis*, EF-Ethylacetate fraction of *M*. *philippensis*, A 4– Active compound of *M*. *philippensis*

### Histological examination

**Fig 2:**
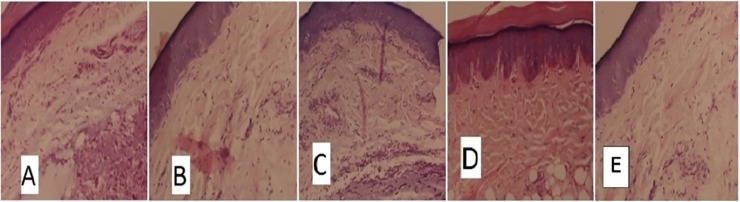
Histological examination of paw sections after 3 h of carrageenan injection. (A) Control group (Normal saline 1ml/kg), (B) Acetylsalicylic acid group(100mg/kg), (C) Ethylacetate fraction group (500mg/kg), (D) Methanol Extract group (500mg/kg), (E) Active Compound 4 group (50mg/kg).

**Fig 3:**
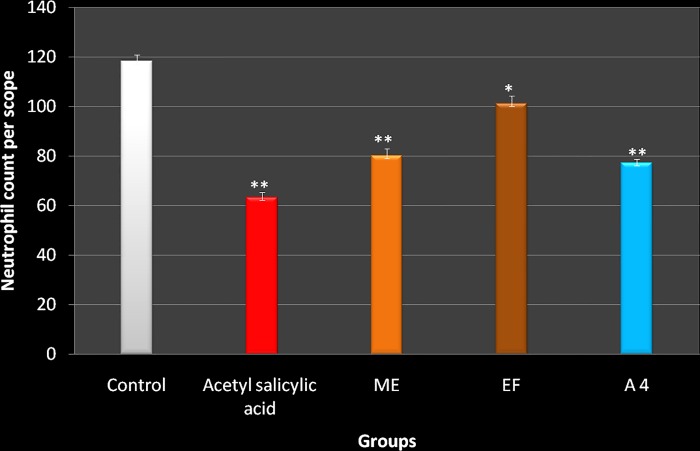
Effect of *M*. *philippensis* leaves on neutrophil infiltration in rat paw after carrageenan injection. Values are expressed as Mean±S.E.M. *indicates p<0.05, ** indicates p <0.001 when compared to the control group Aspirin-100mg/kg, Normal Control- Normal saline 1ml/kg, ME-Methanol extract 500mg/kg, EF-Ethylacetate fraction 500 mg/kg, A 4– Active compound of *M*. *philippensis* (50mg/kg),.

Histological examination of paw sections of rats treated with carrageenan revealed a significant increase in infiltration by neutrophils, tissue injury and accumulation of edema fluid (Fig 2A). Test Groups showed a reduction in carrageenan-induced inflammatory response and there was a significant decrease in the number of neutrophils as compared to the normal control group (p < 0.001) (Figs 2C, 2D, 2E and 3).

## Discussion

Present communication is an effort to find out the anti-inflammatory activity of *M*. *philippensis* and a new flavanoid isolated from it. Carrageenan-induced inflammation is one of the standard models with well characterized molecular mechanisms forthe screening of anti-inflammatory activity of various experimental compounds. It occurs in two phases wherein the early phaseis linked to the production of histamine, leukotrienes, and possibly cyclooxygenase products, while the delayed phase of the carrageenan-induced inflammatory response has been related to neutrophilinfiltration and the production of neutrophil-derived free radicals, such as superoxide, hydrogen peroxide and OH radicals. [22] Paw edema serves as a good indicator in assessing the antiinflammatory activity of test compounds. In the present study, methanol extract and ethylacetate fraction of MP showed significant reduction of edema in both the phases of inflammation but the maximum reduction was observed in the second phase of inflammation.The effect of MP lasted for 3 hours parallel to that of acetylsalicylic acid, indicating a decrease in neutrophilic activity coupled with an antioxidant effect on free radicals.This anti-inflammatory activity couldbe attributed to the presence of various phytochemicals like phenolic compounds, tannins, flavonoids, alkaloids and steroids.[23]Flavonoids present in leaves of MP are known to inhibit mediators of acute inflammation like prostaglandins. [24] Leaves are also shown to be rich in glycosides, sterols and polyphenols which have been reported to possess anti-inflammatory activity by decreasing various mediators of inflammation like prostaglandins, NO, TNF-α, IL-1and IL-6. [25].TNF-α is an important inflammatory mediator which inducean immune response by activating T cells and macrophages leading to secretion of inflammatory cytokines such as IL-1and IL-6. [26]TNF-α is a mediator in carrageenan-induced inflammatory response and induces a further release of kinins and leukotrienes, which are suggested to play an important role in the maintenance of inflammatory response. [27]Therefore we investigated the levels of TNF-α, IL-1 and IL-6 in serum and observed a significant decrease in these inflammatory markers. Also, there is neutrophil infiltration and generation of neutrophil-derived free radicals such as hydroxyl radicals, hydrogen peroxide and superoxide and release of other neutrophil-derived mediators.The histopathology revealed a marked decrease in the cellular infiltration by neutrophils and tissue damage. Lipid peroxidation is due to theattack of free radicals on lipids in cell membranes resulting in accumulation of MDA. Lipid peroxidation not only serves as a marker of tissuedamage *in vivo* but also has been recognized to be the inducer of inflammatory processes. [28]The antioxidant enzymes catalase and GPx plays a crucial role in scavenging H_2_O_2_ and hydroperoxide. [29]MP not only exhibited radical scavenging capacity but also decreased carrageenan-induced lipid peroxidation. We observed a significant increase in catalase and GPx activities,this effect could be due to elevated intracellular antioxidant enzyme activities and decreased oxidative stress in tissues.Excess reactive oxygen species (ROS) tend to cause an oxidative imbalance of the antioxidant system which may result in oxidative stress and inflammation.[30] Given the importance of the oxidative status in the formation of edema, the anti-inflammatory effect exhibited by the drug in this model might be related to its antioxidant properties. The cotton pellet granuloma method has been widely employed to assess the various components of subacute inflammation such as transudative, exudative and proliferative phases. The increase in dry weight of the granuloma measures the proliferative phase due to monocyte infiltration and fibroblast proliferation which is a hallmark of chronic inflammation. [31]Methanol extract, ethylacetate fraction and isolated compound of MP significantly decreased the dry weight of the granuloma when compared to the control group. This anti-inflammatory action may be due to the ability of test compound in reducing the number of fibroblasts and synthesis of collagen and mucopolysaccharide, which are natural proliferative agents in granulation tissue formation. The proinflammatory cytokines like IL-1, IL-6 and TNF-α are powerful chemotactic signals to macrophages and fibroblasts, [32] therefore the inhibition of the same may be responsible for the MP’s anti-inflammatory effect.

## Conclusion

In conclusion, the study suggests that the methanol extract and ethylacetate fraction of MP have potential anti-inflammatory properties. In addition, aflavanone *7*,*4*^*′*^*-Dihydroxy-3*^*′′*^,*3*^*′′*^*-dimethyl -(5*,*6-pyrano-2*^*′′*^*-one)- 8- (3*^*′′′*^,*3*^*′′′*^*-dimethyl allyl)*isolatedfrom the plant also possess anti-inflammatory and antioxidant activity in a dose of 50 mg/kg. The mechanism of anti-inflammatory effect is most probably due to inhibition of chemotaxis of polymorphonuclear cells thereby a decrease in the concentration of proinflammatory mediators like IL-1, IL-6 and TNF-α. Further studies are needed to evaluate the genetic expression of proinflammatory markers to ascertain itsmolecular mechanism.

## Supporting Information

S1 File Extraction and purification of compounds(1–4). Detailed methodology for purification and isolation of compounds
(DOCX)
