# Supplementary material for: Cytokine Attenuation and Free Radical Scavenging Activity of a New Flavanone7,4′-Dihydroxy-3″,3″-Dimethyl -(5,6-Pyrano-2″-One)- 8- (3‴,3‴-Dimethyl Allyl)- Isolated from Mallotus philippensis: Possible Mechanism for Its Anti-Inflammatory Activity
Source: PLoS One. 2016 Dec 12;11(12):e0167294. doi: 10.1371/journal.pone.0167294 (PMC5152807; doi:10.1371/journal.pone.0167294)
Supplement: S1 File — Detailed methodology for purification and isolation of compounds (DOCX) [file pone.0167294.s001.docx]

**Extraction and purification of compounds** (**1-4**)

The air dried powdered kamela, after being defatted with light petroleum ether (60-80^o^C), was exhaustively extracted with different solvent-systems benzene, chloroform, ethyl acetate and methanol respectively. The fractions obtained after eluting the column with different solvent systems in increasing polarity, were subject to crystallization afforded four compounds marked as compounds (**1-4**): Isorotlerin (**1**), 5,7 –Dihydroxy -8- Methyl-6- Prenylflavanone (**2**), 6",6".Dimethylepyrano(2",3,"7,6)-5hydroxyl8, Methylflavanone (**3**) and 7,4′-Dihydroxy-3′′,3′′-dimethyl -(5,6-pyrano-2′′-one)- 8- (3′′′,3′′′-dimethyl allyl)-flavanone (**4**), respectively.

**Chemical Analysis**

The structural elucidation of the isolated compounds (**1-4**) was established on the basis of Elemental analysis, UV, IR, ^1^H NMR, ^13^C NMR and Mass spectral studies, further supported by physical tests for the presence of characteristic functional groups. The analytical results for C and H were within ± 0.3% of the theoretical values. Compound (**4**), was crystallised from CHCl_3_-MeOH as light yellowish solid, m.p.260-261^o^C. Its IR spectrum showed characteristic absorption bands for presence of phenolic OH at 3411 and 3326 cm^-1^, further confirmed from positive ferric chloride test, carbonyl functionality at 1681 and 1623 cm^-1^, and aromatic C=C at 1586 and 1443 cm^-1^. A pair of sharp double doublet at δ 5.58 and 2.83-2.88 integrating for one and two protons is ascribed to H-2 and H-3 hydrogens, respectively. Similarly doublet signals appearing at around δ 7.54 and 7.50 integrating for one proton each was assigned to H-2^′^ and H-6^′^ protons, respectively. Moreover, sharp doublet signals integrating for one proton each at around δ 6.67 and 7.52 was assigned to aromatic protons H-3^′^ and H-5^′^, respectively. A broad band of peaks at around δ 3.63, 2.50, 1.98, 1.57 and 1.59 integrating for 2, 3, 3, 3 and 3 protons was attributed to H- 1^′′^, H-4^′′^, H-5^′′^, H-4^′′′^ and H-5^′′′^, respectively.

^13^C NMR displayed characteristic absorption signals at δ 203.84 and 195.90 corresponding to carbonyl carbons C-2^′′^ and C-4, respectively. Moreover the signals at around δ 162.49 and 157.52 were assigned to hydroxylated carbons C-7 and C-4^′^, respectively. The characteristic signals for aromatic carbons were also observed in the spectrum which is discussed in experimental section.

Finally mass spectroscopy was also in agreement with the proposed structure of the compound **4**, displaying prominent molecular ion peak at m/z 422.47 [M+H^+·^]. Based on the above data, it was concluded that compound **4** is 7,4′-Dihydroxy-3′′,3′′-dimethyl -(5,6-pyrano-2′′-one)- 8- (3′′′,3′′′-dimethyl allyl)-flavanone.

**Compound 4**

We carried out comprehensive investigation of *Mallotus* *philippinensis*, and were able to isolate and characterize four compounds from *Mallotus* *philippinensis*, out of which compound (**4**) is a new compound (***7,4^′^-Dihydroxy-3^′′^,3^′′^-dimethyl -(5,6-pyrano-2^′′^-one)- 8- (3^′′′^,3^′′′^-dimethyl allyl)-flavanone*)** that is isolated from Ethyl acetate-methanol (1:1) fraction.

All the solvents and chemical were purchased from commercial sources (Sigma-Aldrich, Merck and others) and used as received or dried using standard procedures. Melting points were determined on a Kofler apparatus and are uncorrected. Elemental analysis (C, H, N) were conducted using Carlo Erba analyzer model 1108. The IR spectra were recorded on KBr pellets with Interspec 2020 FTIR Spectrometer, values are given in cm^-1^. The UV spectra were recorded with UV VIS-1800 spectrophotometer (Shimadzu). ^1^H and ^13^C NMR spectra were run in CDCl_3_ on a Bruker Avance-II 400 MHz and 100 MHz instrument, respectively. Mass spectra were recorded on a JEOL D-300 mass spectrometer. Thin layer chromatography (TLC) glass plates (20×5 & 50×10) were coated with silica gel (E-Merck G_254_, 0.5 mm thickness) and exposed to iodine vapors to check the purity.
